# Modeling of Fine Root Biomass of Two Dominant Tree Species in the Mixed Broad-Leaved Korean Pine Forest

**DOI:** 10.3390/plants15213395

**Published:** 2026-11-02

**Authors:** Shixiong Wu, Fachang Deng, Wei Zeng, Juntao Liu, Qijing Liu

**Affiliations:** 1 College of Landscape Architecture, Changchun University, Changchun 130022, China; 2 Yuxi Forestry and Grassland Bureau, Yuxi 653100, China; 3 College of Forestry, Beijing Forestry University, Beijing 100083, China; 4 Guangdong Provincial Key Laboratory of Silviculture, Protection and Utilization, Guangdong Academy of Forestry, Guangzhou 510520, China; ljt1120@bjfu.edu.cn

**Keywords:** aboveground (leaves) biomass, fine root surface area, nested regression method, root and leaf traits, root-to-shoot ratio

## Abstract

Quantifying fine root biomass is essential for comprehending forest subsurface ecosystems and predicting changes in plant biomass allocation under heterogeneous soil nutrient conditions. However, little is known about fine root biomass, especially the quantitative analysis of species-specific fine root biomass, due to the destructive, time-consuming, and costly nature of the associated sampling methods. In this study, nested regression was used to estimate the biomass and surface area of fine roots of individual trees of the two main tree species, *Pinus koraiensis* and *Fraxinus mandschurica*, in the primary broad-leaved Korean pine forest. Additionally, the relationship between aboveground traits (leaf biomass and leaf area) and belowground traits (root biomass and surface area of fine roots) was further analyzed. The results show that the fine and total roots of both species were highly fitted to the diameter at breast height with *R*^2^ = 0.92–0.97. The root-to-shoot ratios at the single-plant level were all 0.20. Significant linear relationships existed between leaf biomass and surface area and between fine root biomass and surface area (adjusted *R*^2^ = 0.95, *p* < 0.001). In conclusion, the tight coupling between fine roots and leaves proposed in this study, together with the developed biomass equation, can be used to accurately estimate *Pinus koraiensis* and *Fraxinus mandschurica* biomass at the stand level in primary broad-leaved Korean pine forests.

## 1. Introduction

Fine roots (diameter ≤ 2 mm or terminal roots of 1st to 3rd order) are highly physiologically active and sensitive parts of the root system and serve as the primary organs for water and nutrient absorption [1]. Although fine roots account for only 0.5–10% of forest biomass [2], due to their rapid growth and turnover rates, they play a crucial role in subsurface carbon (C) and nutrient cycles in terrestrial ecosystems and may contribute 33–75% of the net primary productivity of forests [3]. Previous research has indicated that fine roots constitute a crucial component of belowground carbon flux in forest ecosystems, primarily through nutrient and water uptake, which helps sustain plant functioning and returning carbon back to the soil via decomposition [4]. Currently, sampling and measuring fine roots is exceedingly challenging. Their short lifespan and small diameter complicate accurate biomass estimation, making precise quantification of fine root biomass essential for studying forest carbon storage.

Over recent decades, despite the inherent difficulties, substantial research has been conducted on fine root production and turnover [5]. The main methodologies include direct measurements such as soil coring and minirhizotron techniques, as well as indirect estimations using allometric equations and root-to-shoot ratio [6,7]. These methods involve direct root sampling from soil cores or blocks, employing the allometric relationship between root biomass and tree size parameters, or utilizing the ratio of belowground to aboveground biomass at the stand scale. Using allometric equations to estimate root biomass can enhance accuracy, and if the sample size is substantial and represents a range of tree ages and sizes, the results can be extrapolated to other stands [8]. However, there is currently no universally applicable allometric equation for estimating belowground biomass in temperate broadleaf–pine forests. The root-to-shoot ratio method can estimate belowground biomass from stand-scale aboveground biomass, but factors such as stand age, soil conditions, and precipitation have been shown to influence the root-to-shoot ratio, highlighting data gaps and the limitations of precise biomass estimation [9,10].

The temperate forests in northeastern China account for one-third of the forest area and standing stock in the country and play a crucial role in national carbon budgets [11]. However, due to the difficulties in root sampling, the belowground carbon pool and fluxes in these forests have not been quantified [12,13]. *Pinus koraiensis* and *Fraxinus mandschurica* are the main species representing the climax vegetation type of northeast China’s eastern mountainous area. Extensive research has been conducted on these two species’ plantation, focusing on fine root biomass, morphology, senescence, lifespan, and turnover [14,15]. Yet, studies of fine roots in primary forests of these two species are limited, particularly those estimating fine root biomass. Mei et al. [16] found that the fine root biomass in a 20-year-old *F. mandschurica* plantation was 181.96 g m^−2^. Over the past decade, domestic research employing minirhizotron technology for fine root studies has made substantial progress. Shi et al. [17] used minirhizotrons to study fine root growth, mortality, and turnover in larch and *F. mandschurica* plantations, revealing that the annual production and mortality of larch fine roots were significantly lower than those of *F. mandschurica*. These methods are intricate and time-consuming, whereas this study utilizes a nested regression approach to estimate the fine root biomass of *F. mandschurica*. This method has successfully developed models for canopy biomass estimation of 10 tree species in broadleaf–pine forests and fine root biomass estimation of *Larix gmelinii (Rupr.) Kuzen* and *Pinus tabuliformis* [18,19,20]. It minimizes disturbance to soils and trees and supports non-destructive sampling, avoiding destructive whole-tree harvesting. The objectives of this study were (1) to estimate the root-to-shoot ratio and biomass of *P. koraiensis* and *F. mandschurica* at the stand level based on the belowground biomass and (2) to explore the relationship between leaf biomass and fine root biomass.

## 2. Results

### 2.1. Allometric Growth Equations for Different Root Indicators

Segmented power function fitting was applied to root axis and segment volume, fine root biomass, surface area, and total fine root length. The model parameters and statistical results are presented in Table 1. All models were statistically significant (*p* < 0.001) and performed well, with $R_{\mathrm{adj}}^{2}$ values ranging from 0.84 to 0.99. Based on these models, the volume of root axes and segments can be used to estimate the coarse root biomass, fine root biomass, and surface area of individual trees by multiplying by the density of coarse and fine roots, respectively.

### 2.2. Model Verification

**Figure 1:**
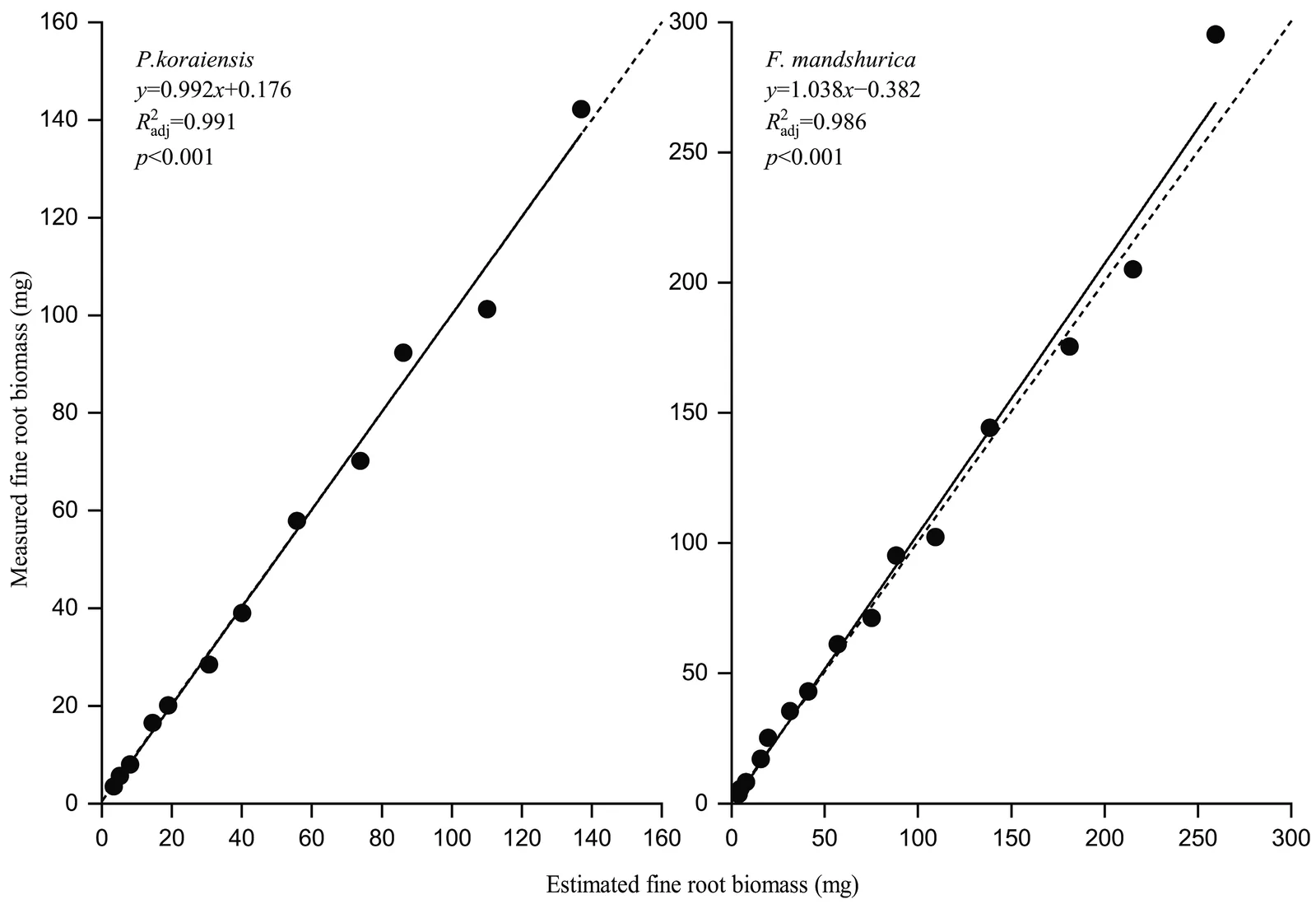
Relationship between measured fine root biomass and estimated fine root biomass of *P. koraiensis* and *F. mandshurica.* The solid line in the fitting relationship indicates the estimated and measured value fitting line, and the dashed line indicates the *y* = *x* reference line.

The fine root biomass of *P. koraiensis* and *F. mandshurica* was measured using independent 12 and 15 intact root sections, respectively (Figure 1). The root diameter distributions for the two species ranged from 2.79 to 38.13 mm and from 2.84 to 45.21 mm, respectively. The nested regression method was used to calculate the estimated values for the model. The reliability of the model was determined based on the fitting performance and test results of the measured and estimated values. Two linear regression analysis results indicate that the estimated values are consistent with and highly correlated to the measured values ($R_{\mathrm{adj}}^{2}=\;0$.99). A *t*-test demonstrated no significant difference between the estimated and measured values at the 0.05 significance level, confirming the model’s reliability.

### 2.3. Model Equations for Root Biomass and Fine Root Surface Area

Allometric equations were used to fit the relationships between total root biomass, fine root biomass, fine root surface area, and DBH. Both results show that total root biomass, fine root biomass, and fine root surface area were all significantly positively correlated with DBH (*p* < 0.001). The adjusted coefficients of determination were high, all greater than 0.94, indicating a strong fitting performance (Table 2).

### 2.4. Relationship Between Root Biomass and Aboveground Traits

**Figure 2:**
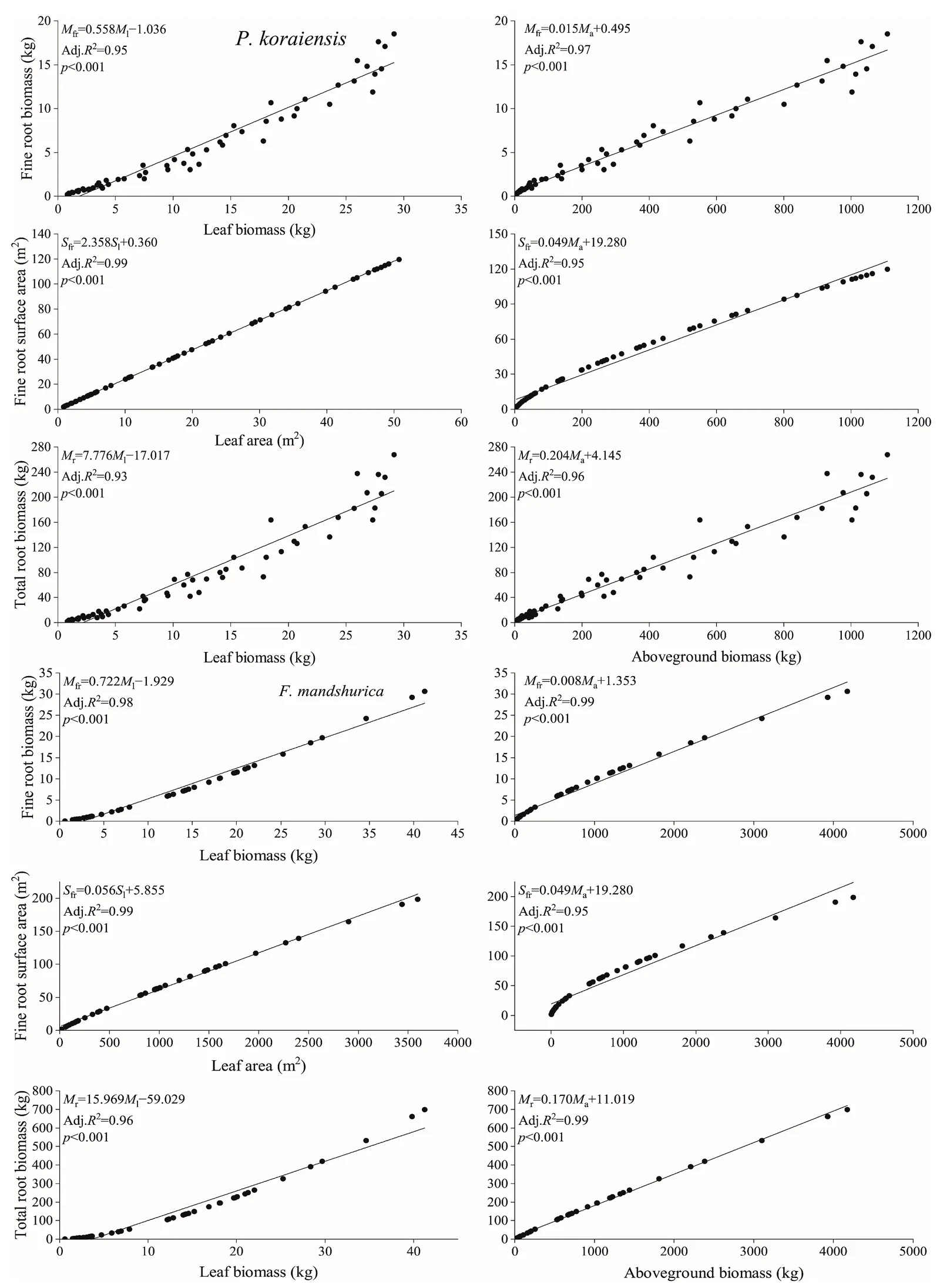
Regression relationships between root and aboveground traits in components at individual tree level for *P. koraiensis* and *F. mandshurica.*

At the individual tree level, there was a highly significant (*p* < 0.001) linear positive correlation between the fine root biomass of *P. koraiensis* and *F. mandschurica* and both leaf biomass ($R_{\mathrm{adj}}^{2}$^2^ values of 0.95 and 0.98, respectively) and aboveground biomass ($R_{\mathrm{adj}}^{2}$^2^ values of 0.97 and 0.99, respectively), with good fitting performance, indicating that the biomass allocated to leaves was greater than that allocated to fine roots (Figure 2). Additionally, a strong positive linear relationship was observed between the fine root surface area and both leaf area ($R_{\mathrm{adj}}^{2}$ values of 0.99 and 0.99, respectively) and aboveground biomass ($R_{\mathrm{adj}}^{2}$ values of 0.95 and 0.95, respectively). Total root biomass was closely related to leaf biomass ($R_{\mathrm{adj}}^{2}$ values of 0.93 and 0.96, respectively) and aboveground biomass ($R_{\mathrm{adj}}^{2}$ values of 0.96 and 0.99, respectively).

### 2.5. Stand-Level Biomass Estimation

At the stand level, equations based on DBH were used to predict the total root biomass, fine root biomass, and surface area of *P. koraiensis* and *F. mandshurica* within a 25 ha broad-leaved Korean pine forest (Table 3). Results indicate that the stand density was 496 trees·ha^−1^, with *P. koraiensis* accounting for 92 trees·ha^−1^ and *F. mandschurica* accounting for 21 trees·ha^−1^. Notably, the root-to-shoot ratio of two species was 0.20. For *P. koraiensis*, the total biomass and root biomass were 91.71 t·ha^−1^ and 16.67 t·ha^−1^, respectively. The fine root biomass was 1.21 t·ha^−1^, accounting for 7% of the total root biomass. Additionally, the leaf biomass density was 3.70 t·ha^−1^, approximately 3 times that of the fine root biomass. The surface area of fine root was 8727.63 m^2^·ha^−1^. For *F. mandschurica*, the total biomass and root biomass were 78.67 t·ha^−1^ and 11.35 t·ha^−1^, respectively. The fine root biomass was 0.50 t·ha^−1^, accounting for 4% of the total root biomass. Additionally, the leaf biomass density was 0.70 t·ha^−1^, approximately 1.4 times that of the fine root biomass. The surface area of fine root was 3329.82 m^2^·ha^−1^.

## 3. Discussion

### 3.1. Precision of Nested Regression Method

Research on tree root systems still faces numerous challenges regarding methodology and accuracy. During the excavation and dissection process, it is inevitable to encounter broken roots due to human activities or obstacles like rocks, making it difficult to obtain complete fine root anatomical data. In this study, to address the issues of broken roots during sampling and measurement of fine roots and large amounts of fresh fallen root systems, we assumed that roots of the same diameter had identical length, surface area, and volume. By grafting roots of similar diameters, we corrected the broken root diameter and length data [18]. Specifically, the diameter at the broken root was measured, and a root diameter and length of the same or similar size were substituted in calculations to achieve complete fine root data. Evaluating belowground biomass is a labor-intensive and time-consuming task. Root loss and separation from soil during excavation pose significant challenges for accurate biomass estimation [21]. The nested regression method used in this study addresses these issues by efficiently obtaining the entire root biomass, including fine roots, of individual trees without the cumbersome weighing process, thus minimizing workload, especially when time or labor is limited [22]. This approach also significantly reduces root exposure time to the atmosphere, preserving the original moisture content of the roots and ensuring accurate measurements. When recording coarse root diameters, only the necessary parts were exposed without destructively cutting the sample trees. The accuracy of this method depends on the construction of the root axis regression model, as it serves as the foundation for the stepwise development of successive root branch models. Due to the cumulative nature of errors, even minor deviations in the root axis model may result in significant inaccuracies in the final model. As shown in Figure 1, the root axis accuracy for both tree species exceeds 98.6%, ensuring the reliability of the regression models. Moreover, this method has been validated in previous studies, including research on larch (*Larix* spp.) [19]. In addition, we reviewed the existing literature and found that empirical fine root biomass data suitable for validating allometric equations are currently unavailable for primary broad-leaved Korean pine forests. We acknowledge that independent validation against in situ measured whole tree fine root biomass is still lacking, and we expect to conduct such validation when field conditions permit in future work.

### 3.2. Fine Root Biomass at the Stand Level

The nested regression method employed in this study has been reported in the literature for root system and fine root biomass models of different tree species [18,19]. In this study, the equations for estimating *P. koraiensis* and *F. mandschurica* root biomass were developed from simulations based on 58 and 40 large trees of varying DBH, respectively, meeting the crucial sample size requirement for equation development [23]. Thus, the simulated belowground biomass equations are reliable for further biomass estimation of these two species. Additionally, most existing biomass models for these two species were developed for pure plantations or without considering fine root biomass, which could result in significant biases [24,25]. Therefore, the newly developed biomass models are expected to provide accurate biomass predictions and can be applied to species (*P. koraiensis* and *F. mandschurica*) in the mixed broad-leaved Korean pine forest. In this study, total root biomass and fine root biomass were significantly correlated with DBH, enabling stand-level root biomass estimation. Combining data from a 25 ha broad-leaved Korean pine forest, for *P. koraiensis*, the fine root surface area was 8727.63 m^2^·ha^−1^, total biomass was 91.71 t·ha^−1^, root biomass was 16.67 t·ha^−1^, and fine root biomass was 1.21 t·ha^−1^, accounting for approximately 7.25% of the total root biomass. This is slightly lower or higher compared to previous studies on the same species. For example, Li and Han [26] reported that the fine root and belowground biomasses in the mixed broad-leaved Korean pine plantation were about 2.07 ± 0.18 and 14.2 ± 1.42 t·ha^−1^, respectively. For *Fraxinus mandschurica*, the fine root surface area was 3329.82 m^2^·ha^−1^, total biomass was 78.67 t·ha^−1^, root biomass was 11.35 t·ha^−1^, and fine root biomass was 0.50 t·ha^−1^, accounting for approximately 4.41% of the total root biomass. This is slightly lower compared to previous studies on the same species. For example, Xie et al. [27] reported fine root biomasses in *F. mandschurica* plantations ranging from 279 to 283 g·m^−2^, and Mei et al. [16] reported 290.77 g·m^−2^ for a 17-year-old *F. mandschurica* plantation. Zhou et al. [28] found fine root biomasses of 487.8 g·m^−2^ in a 50-year-old natural secondary forest. The main reasons for the differences in fine root biomasses of the two species in different stands may be attributed to variations in climatic conditions [29], species composition [30], stand density, forest age [31], and fine root collection methods. Overall, the results are comparable. Nevertheless, many fine roots are broken and retained in the soil during tree uprooting, and root plates poorly capture deep root fractions. Consequently, fine root biomass derived from wind-thrown trees in this study is likely underestimated.

At the stand level, Guo et al. [32] reported fine root biomass of 4.49 t·hm^−2^ in the broad-leaved Korean pine forest within the Changbai Mountain region, suggesting that the fine root biomass of *P. koraiensis* (1.21 t·ha^−1^) accounts for 27% and *F. mandschurica* (0.49 t·ha^−1^) accounts for 11% of the total fine root biomass of the forest. At the individual plant level, although the fine root biomass was greater for *F. mandschurica* than *P. koraiensis*, this result was reversed at the stand level, which may be related to stand density, with *P. koraiensis* as the main establishment species in the top vegetation forest (92 trees·ha^−1^), and *F. mandschurica* as a companion species (21 trees·ha^−1^). This is in line with the higher fine root biomass of species with high species abundance and the fact that higher species abundance can more fully utilize the soil space and soil resources [33,34]. Our study also found that the root-to-shoot ratio of both *P. koraiensis* and *F. mandschurica* was 0.2, lower than the 0.28 ± 0.09 ratio reported by Dong [35] in the northeastern forest region but within the error range. This slight difference may be due to the variation in the forest stands surveyed. Other widely used root-to-shoot ratios are between 0.2 and 0.5 for temperate forests, according to Mokany et al. [36]. This metric is commonly used to estimate belowground biomass in large-scale forest biomass assessments, though the ratio varies depending on environmental and forest stand conditions.

### 3.3. Relationship Between Fine Root Biomass and Leaf Biomass

Fine roots in many regards are the belowground analogs to leaves, as both are important sites for material distribution and exchange between above and below ground [37]. The plant economics spectrum hypothesis suggests a strong covariation between leaf and root traits [38,39]. Our results confirm this hypothesis, showing significant linear relationships between *P. koraiensis* and *F. mandschurica* root biomass, fine root biomass, and leaf biomass, consistent with the linear relationship between fine root and leaf biomass found in coniferous species. In a study by Cermak and Nadezhdina [40], the absorptive root surface area of nearly 500 trees of 11 woody plants across different ages, sizes, and growth conditions showed a linear relationship with the cross-sectional area at breast height, which was proportional to leaf area. This implies a linear relationship between individual tree absorptive root surface area and leaf area, consistent with our finding of a significant positive correlation between fine root surface area and leaf area and indicating that trees maintain a cohesive hydraulic structure and function from fine roots to leaves. Additionally, observed root–leaf covariation may be partly driven by shared tree-size effects and cannot be interpreted as direct evidence for functional balance or the plant economics spectrum without further size-corrected analysis.

Previous investigations on biomass allocation patterns have demonstrated a positive correlation between aboveground biomass (AGB) and belowground biomass [41]. Several further studies have indicated that greater fine root biomass (FRB) sustains higher AGB, implying strong coupling between tree growth and root absorption [42], and Finér et al. [43] documented significant increases in fine root biomass with rising basal area, a proxy for aboveground biomass, across broad geographic gradients. Nevertheless, other work argued that fine root biomass, or fine root proportion, correlates more closely with leaf biomass than with aboveground biomass [37]. Here, we tested the hypothesis that fine root biomass (FRB) exhibits a positive allometric relationship with aboveground biomass (AGB) and leaf biomass, with a strong association expected for leaf biomass (Hypothesis 1). This hypothesis is based on the premise that greater AGB generally reflects a faster growth strategy under favorable climatic conditions, which is accompanied by increased carbon allocation to both above- and belowground organs. Such coordinated biomass allocation may explain the positive relationships previously reported between AGB and FRB [43], as well as between leaf biomass and FRB [37]. Fine root and leaf phenology are commonly asynchronous across temperate forest ecosystems. As a result, fine root biomass and its linkage to leaf biomass are strongly time dependent and sensitive to the timing of root sampling. Previous studies have shown that fine root biomass typically displays bimodal seasonal peaks, occurring in late spring and early autumn in synchrony with periods of active plant growth. To minimize the influence of seasonal fluctuations, root sampling in the present study was conducted during the relatively stable summer period, thereby providing a more representative estimate of standing fine root biomass. Nevertheless, sampling conducted at different phenological windows may therefore yield divergent estimates of fine root biomass and alter the observed linear relationship between fine roots and leaf biomass. We estimate that approximately one-quarter of the total plant carbon is stored belowground, stressing the importance of root systems for the terrestrial carbon sink [44]. Because estimates of root biomass are commonly derived from aboveground biomass through allometric scaling relationships, uncertainties in AGB estimation inevitably propagate into belowground carbon estimates. Therefore, improving the accuracy of aboveground biomass assessments and refining predictive models of belowground biomass allocation are essential for reducing uncertainty in estimates of total plant biomass, ecosystem carbon stocks, and ecosystem functioning under both current and future climate conditions.

## 4. Materials and Methods

### 4.1. Study Area

The experimental site for this study is primarily located in the broadleaf–pine forest zone within the Changbai Mountain Nature Reserve on the northern slope (41°42′45″–42°45′18″ N, 127°33′30″–128°16′48″ E), at an altitude of approximately 800 m. The annual average temperature ranges from 3 °C to 8 °C, with an effective accumulated temperature of 2500 °C to 3000 °C. The annual sunshine duration is less than 2400 h, and the frost-free period is 90 days to 150 days. The annual average precipitation ranges from 700 to 1400 mm, with 60%~80% of the precipitation occurring from June to August. Due to Changbai Mountain being a dormant volcano, differences in geological features, soil, vegetation, and climate have resulted in distinct soil types and a clear vertical distribution. The climate within the forest is cold and humid, with a short growing season, leading to slow decomposition and significant accumulation of organic matter. The soil is peaty, with a thin surface layer and indistinct soil horizons. In 2022, a 500 m × 500 m broadleaf–pine forest plot was established, where all trees with a diameter at breast height (DBH) greater than 5 cm were measured. Tree identification numbers, species names, and DBH measurements were recorded.

### 4.2. Sampling and Measurement

Within the stand, 58 stems of *P. koraiensis* (*DBH_min_* = 5.1 cm, *DBH_max_* = 47.5 cm) and 40 stems of *F. mandschurica* (*DBH_min_* = 3.5 cm, *DBH_max_* = 67.9 cm) were randomly selected and excavated to the level of measurable primary roots during July to August of 2020 and 2021, respectively. The diameters of all primary roots at the base of the tree stumps were measured at the roundest part using a vernier caliper or DBH tape, measuring only the primary root diameters. The DBH of the sampled trees was also recorded. Additionally, fresh *P. koraiensis* and *F. mandschurica* windthrown trees caused by typhoons were located, and their roots were dissected into different diameter classes based on a nested principle. The basal diameter and axial length of the graded roots were measured. For roots with an axial length less than 30 cm, volume was calculated using the cone formula, while longer roots were divided into segments of 30~50 cm, and diameters at both ends were measured. We sampled 15 *P. koraiensis* and 13 *F. mandschurica* fresh windthrown trees opportunistically over multiple years. The DBH of sampled *P. koraiensis* ranged from 14.6 to 31.4 cm, while that of *F. mandschurica* ranged from 23.7 to 53.8 cm. This classification and dissection of roots from numerous windthrown trees provided a dataset of all primary root diameter ranges for the 58 and 40 sampling trees. These data were then used to calculate the volume, surface area, and total length of root axes and root segments, fitting equations for the relationship between root diameter and volume, surface area, and total length. The following two formulas use the mean sectional area method to calculate the volume of root axes and the surface area of fine roots.

(1) $$V=\frac{\pi l}{8}\left[d_{0}^{2}+d_{n}^{2}+2\sum_{i=1}^{n-1}d_{i}^{2}\right]+\frac{\pi }{12}d_{n}^{2}l^{\prime }$$

(2) $$S=\frac{\pi l}{2}\left[d_{0}+d_{n}+2\sum_{i=1}^{n-1}d_{i}\right]+\frac{\pi }{2}d_{n}l^{\prime }\;$$

where *V* and *S* represent the root axis volume and surface area, respectively. *d*_0_, *d_i_*, and *d_n_* are the diameters of the root base, top, and bottom ends of each segment, respectively. *l* and *l’* denote the lengths of the root segments and root tips. In the field, relatively intact and variously sized fresh roots were excavated.

Owing to barriers including bedrock, fine roots are rarely recovered completely without breakage or missing portions. When full excavation was not achievable, diameters were measured at the fracture positions of incompletely extracted roots. We applied a root splicing approach to pair fractured roots with discrete root segments sharing identical diameter values at the break points. Coarse roots were cut into relatively uniform sections using a saw, while fine roots were trimmed using pruning shears. The root volume was measured on the same day using the water displacement method for coarse roots and the cylindrical volume formula for fine roots. Fresh weight was determined using an electronic balance with 0.0001 g precision. The samples were then dried in a 60 °C constant-temperature oven. Dry weight was repeatedly measured until a constant weight was achieved, which was recorded as the dry weight. Root density was calculated as the dry weight divided by the fresh volume of the root sample. During root dissection, various grades of intact fine roots of sample trees were selected, cleaned, and dissected in the laboratory. Fine roots at the terminal ends were defined as first-order roots. The intersection of two first-order roots formed a second-order root, and so forth; the intersection of two second-order roots formed a third-order root. The different grades of fine roots were examined under a 40× dissecting microscope to measure their diameter and length. Table 4 presents these descriptive statistics for fine root variables.

### 4.3. Model Fitting

The volume of the root system was calculated using a progressive reverse-order method. The volume of lateral roots on second-order root segments (i.e., first-order root axes) was summed, then added to the volume of the second-order root axis itself to obtain the volume of the second-order root segment. A regression equation was established using these second-order root segment volume data. Similarly, the total volume of the third-order root axis and its lateral roots was calculated using the fitted model to determine the volume of the third-order root segment. This process expanded the root diameter range progressively until reaching the primary root segment. Although the final model is based on root axes and segments, it applies to all root diameters within the existing range, regardless of hierarchical order, and is key to developing the root–shoot biomass and DBH model. By substituting the corresponding root diameter into the relevant equation, the volume of root segments of specific diameters can be estimated. Our nested regression assumes that roots of identical diameter share consistent geometric properties, ignoring potential variation from root order, soil depth and tree size.

For fine roots, calculations are limited to diameters within 2 mm, and accumulation does not continue beyond this. However, the progressive fitting process is carried out until all primary root segments on the tree stump are included. The total values for fine root indices of an individual tree can be obtained by summing all fine roots on the primary root segments. Multiplying the average root density by the root segment volume model converts it into a root segment biomass model. By substituting all primary root diameters of an individual tree stump into the corresponding diameter range equations, the biomass of each primary root segment can be calculated. Summing these values yields the total root biomass of the individual sampling tree. In this manner, the root biomass of the sampling trees can be determined, and a model fitting root biomass to DBH can be developed. Root branching classification refers to the schematic diagram in Figure 1 from Meng et al. [19].

### 4.4. Model Evaluation

In this study, the adjusted coefficient of determination (Adj. *R*^2^) was used to evaluate the model’s goodness of fit. The calculation formula is as follows:

(3) R2=1−∑i=1n(yi−y^i)2∑i=1n(yi−y¯)2

(4) $$Adj.R^{2}=1-(1-R^{2})\frac{n-1}{n-p}\;$$

where *y_i_* is the observed value, y^i is the predicted value, $\overset{¯}{y}$ is the mean of the observed data values, *n* is the total number of data points used for model fitting, and *p* represents the number of parameters included in the model.

### 4.5. Data Analysis

Regression analyses were conducted using Origin 2022b and R 4.0.3 to develop models for root biomass, fine root biomass, and surface area. All equations were fitted using linear models, power function models, and multivariate stepwise regression. The most suitable models were selected based on the coefficient of determination (*R*^2^) and F-tests. Due to the wide range of root base diameters, segmented equations were used to fit areas with significant volume changes, reducing error and improving model accuracy. Aboveground biomass equations for different components of sampling trees were taken from Xu et al. [20].

## 5. Conclusions

Using the nested regression method, we established equations linking DBH with belowground root biomass, fine root biomass, and fine root surface area for individual *P. koraiensis* and *F. mandschurica* trees, yielding significant allometric growth models. These models are crucial for understanding tree growth processes, particularly belowground biomass estimation. They play a vital role in estimating stand-scale belowground root metrics and are significant for further understanding the underground carbon pool in the mixed broad-leaved Korean pine forest.

## Figures and Tables

**Table 1:** Estimation of regression parameters of different root indicators for *P. koraiensis* and *F. mandshurica* in relation to root diameter. All equations follow the form of *y* = *ax^b^*, where *y* represents volume (mm^3^), biomass (mg), surface area (mm^2^), or root length (mm); *x* represents root diameter (mm); *a* and *b* are statistics and estimates of the power function model.

	Component	Diameter Range (mm)	*a*	*b*	$R_{\mathrm{adj}}^{2}$	*n*	*p* Value
*P. koraiensis*	Volume of main root branch	0 < x ≤ 0.22	1913.08	5.9	0.98	837	<0.001
		0.22 < x ≤ 2	122.17	3.49	0.96	724	<0.001
		2 < x ≤ 20	213.62	2.02	0.95	362	<0.001
		20 < x ≤ 338	239.81	2.16	0.88	185	<0.001
	Volume of fine root branch	0 < x ≤ 0.22	1913.08	5.9	0.98	837	<0.001
		0.22 < x ≤ 1.84	739.5	3.86	0.95	512	<0.001
		1.84 < x ≤ 12	1612.44	2.84	0.91	271	<0.001
		12 < x ≤ 338	9163.05	1.58	0.86	97	<0.001
	Fine root biomass	0 < x ≤ 0.22	2135.71	6.47	0.99	837	<0.001
		0.22 < x ≤ 2.37	109.54	3.91	0.98	720	<0.001
		2.37 < x ≤ 18	844.33	1.34	0.95	362	<0.001
		18 < x ≤ 338	807.29	1.41	0.88	97	<0.001
	Fine root surface area	0 < x ≤ 0.22	6172.11	6.61	0.99	746	<0.001
		0.22 < x ≤ 1.34	1632.92	2.52	0.96	487	<0.001
		1.34 < x ≤ 11.42	2457.03	1.94	0.91	139	<0.001
		11.42 < x ≤ 338	4974.52	1.49	0.84	56	<0.001
	Total length of fine root	0 < x ≤ 0.22	9382.27	4.81	0.99	816	<0.001
		0.22 < x ≤ 1.88	1873.17	4.26	0.95	431	<0.001
		1.88 < x ≤ 22	976.08	1.33	0.86	286	<0.001
		22 < x ≤ 338	415.22	1.67	0.87	64	<0.001
*F. mandshurica*	Volume of main root branch	0 < x ≤ 0.30	29.63	2.70	0.98	724	<0.001
		0.30 < x ≤ 0.70	58.46	2.56	0.96	420	<0.001
		0.70 < x ≤ 2.00	67.04	2.74	0.95	253	<0.001
		2 < x ≤ 20	692.14	2.16	0.93	113	<0.001
		20 < x ≤ 200	57.76	2.76	0.89	75	<0.001
	Volume of fine root branch	0 < x ≤ 0.30	29.63	2.70	0.98	724	<0.001
		0.30 < x ≤ 0.70	99.16	2.49	0.96	351	<0.001
		0.70 < x ≤ 2.0	137.72	2.87	0.94	162	<0.001
		2 < x ≤ 20	1276.75	2.16	0.91	77	<0.001
		20 < x ≤ 200	173.29	2.76	0.87	36	<0.001
	Fine root biomass	0 < x ≤ 0.30	16.50	2.79	0.98	724	<0.001
		0.30 < x ≤ 0.70	49.26	2.49	0.96	420	<0.001
		0.70 < x ≤ 2.00	68.41	2.87	0.96	253	<0.001
	Fine root surface area	0 < x ≤ 0.30	191.09	2.00	0.95	724	<0.001
		0.30 < x ≤ 0.70	916.38	2.07	0.94	351	<0.001
		0.70 < x ≤ 2.00	1278.01	2.82	0.92	162	<0.001
	Total length of fine root	0 < x ≤ 0.30	283.59	2.03	0.98	724	<0.001
		0.30 < x ≤ 0.70	1029.59	2.06	0.94	351	<0.001
		0.70 < x ≤ 2.00	1458.07	2.92	0.92	162	<0.001

**Table 2:** The power function model of each component of *P. koraiensis* and *F. mandshurica.*

Biomass	Species	Equation	$R_{\mathrm{adj}}^{2}$	*p* Value
Total root biomass (kg)	*P. koraiensis*	*W*_R_ = 0.0292 *D* ^2.322^	0.96	<0.001
	*F. mandshurica*	*W*_R_ = 0.0678 *D* ^2.191^	0.94	<0.001
Fine root biomass (kg)	*P.koraiensis*	*W*_fr_ = 0.00334 *D* ^2.201^	0.97	<0.001
	*F. mandshurica*	*W*_fr_ = 0.01002 *D* ^1.903^	0.98	<0.001
Fine root surface (m^2^)	*P.koraiensis*	*S*_fr_ = 0.021 *D* ^1.127^	0.95	<0.001
	*F. mandshurica*	*S*_fr_ = 0.317 *D* ^1.527^	0.98	<0.001

**Table 3:** Each index of *P. koraiensis* and *F. mandshurica* in the mixed broad-leaved Korean pine forest.

Indicators	*P. koraiensis*	*F. mandshurica*
Stand density (stem·ha^−1^)	496	496
Stem	92	21
Average DBH (cm)	39.7	57.1
Total biomass (t·ha^−1^)	91.71	78.67
Leaf biomass (t·ha^−1^)	3.70	0.70
Root biomass (t·ha^−1^)	16.67	11.35
Fine root biomass (t·ha^−1^)	1.21	0.50
Surface area of fine roots (m^2^·ha^−1^)	8727.63	3329.82
Root-to-shoot ratio	0.20	0.20

**Table 4:** Morphological attribute values (mean ± standard error) of fine roots between each adjacent root sequence at the final stage of *P. koraiensis* and *F. mandshurica.*

Species	Root Order	Diameter (mm)	Root Length (mm)	SRL (m·g ^−1^)	Biomass (mg)	Surface Area (mm^2^)	Sample
*P. koraiensis*	1	0.22 ± 0.01	6.54 ± 0.14	55.97 ± 1.52	0.12 ± 0.008	4.60 ± 0.03	200
	2	0.37 ± 0.02	27.31 ± 0.90	18.98 ± 0.47	1.36 ± 0.02	31.92 ± 0.58	150
	3	0.51 ± 0.02	56.76 ± 2.04	10.20 ± 0.40	5.57 ± 0.13	91.19 ± 3.15	100
	4	0.89 ± 0.06	105.44 ± 2.38	3.35 ± 0.16	31.77 ± 1.87	295.82 ± 5.81	80
	5	1.96 ± 0.21	287.70 ± 9.57	0.65 ± 0.04	416.63 ± 5.48	1706.65 ± 12.47	60
*F. mandshurica*	1	0.23 ± 0.03	13.73 ± 0.43	52.23 ± 1.98	0.27 ± 0.09	9.69 ± 0.50	200
	2	0.45 ± 0.09	45.61 ± 1.34	14.27 ± 7.77	4.07 ± 0.17	67.18 ± 2.33	150
	3	0.92 ± 0.09	65.92 ± 2.91	6.79 ± 4.98	16.11 ± 0.73	157.44 ± 3.64	100

## Data Availability

The original contributions presented in this study are included in the article. Further inquiries can be directed to the corresponding authors.
